# Retinal structure in Leber’s congenital amaurosis caused by *RPGRIP1* mutations

**DOI:** 10.1038/s41439-019-0064-8

**Published:** 2019-06-27

**Authors:** Daisuke Miyamichi, Sachiko Nishina, Katsuhiro Hosono, Tadashi Yokoi, Kentaro Kurata, Miho Sato, Yoshihiro Hotta, Noriyuki Azuma

**Affiliations:** 1 Department of Ophthalmology, Hamamatsu University School of Medicine, Shizuoka, Japan; 2 Department of Ophthalmology and Laboratory for Visual Science, National Center for Child Health and Development, Tokyo, Japan

**Keywords:** Genetic testing, Targeted gene repair

## Abstract

This study aimed to evaluate retinal structure in the early stage of Leber’s congenital amaurosis (LCA) caused by *RPGRIP1* mutations. Four patients from two families were included. Case 1 was a 13-year-old girl, cases 2 and 3 were 7-year-old monozygotic twin brothers of case 1, and case 4 was a 17-year-old boy. Comprehensive ophthalmic examinations were performed, including visual acuity measurements, perimetry, electroretinography (ERG), and optical coherence tomography (OCT). To identify potential pathogenic mutations, 74 genes known to cause retinitis pigmentosa or LCA were assessed using targeted next-generation sequencing. OCT showed photoreceptor outer nuclear layer (ONL) thinning in all patients. The lamellar structure was retained in all patients, whereas the ellipsoid zone was extinguished in cases 1, 2, and 3. In case 4, the ellipsoid zone was maintained at 9 years of age but became blurred at 17 years of age. In case 1, OCT indicated slight photoreceptor ONL thinning during the period between 7 and 11 years of age. Mutation analysis revealed *RPGRIP1* mutations as the cause for autosomal recessive LCA in all patients. Photoreceptor ONL on OCT is relatively well preserved in the early stage of LCA caused by *RPGRIP1* mutations.

## Eye disease: Mutations affecting retinal structure

Researchers in Japan have characterized the early stages of the inherited retinal disease Leber’s congenital amaurosis (LCA), raising hope that gene therapy could help before the disease progresses too far. LCA results in early-onset blindness or severe visual impairment and has been linked with several genes, including *RPGRIP1*. Daisuke Miyamichi from Hamamatsu University School of Medicine, Japan, and coworkers conducted ophthalamic tests on four young patients with *RPGRIP1* mutations. In all four cases, they found thinning of the photoreceptor outer nuclear layer, a layer of the retina. The outer nuclear layer progressively thinned in consecutive samples taken from the same patient at different ages, and was better retained in the younger patients. Taken together, these findings suggest that gene therapy to correct *RPGRIP1* mutations could be effective if carried out in early childhood.

## Introduction

Leber’s congenital amaurosis (LCA) is one of the most severe forms of inherited retinal dystrophies. It is characterized by early-onset blindness or severe visual impairment during the first year of life, a specific behavior known as Franceschetti’s oculo-digital sign, congenital nystagmus, a sluggish pupillary reaction, and extinguished or severely reduced rod and/or cone responses on electroretinography (ERG)^1^. LCA is diagnosed primarily on the basis of severely impaired vision with some clinical characteristics in infancy, and genetic testing is known to play a role in confirmation of the diagnosis.

LCA is generally inherited as an autosomal recessive trait, although some families with autosomal dominant inheritance have been described^2^. The condition has been reported in many countries, and its worldwide incidence is 1–2 in 80,000 individuals^3,4^. Twenty-five causative genes have been identified^5^, including the *RPGRIP1* gene, which encodes retinitis pigmentosa GTPase regulating interacting protein 1 and contributes to approximately 5% of cases of LCA^6^.

Several genotype–phenotype correlations that allow for the prediction of the gene from the phenotype characteristics have been confirmed. Koenekoop et al. described the correlation between gene defects and the natural history of visual function in patients with LCA. *RPGRIP1* mutations are a degenerative type of mutation that are associated with a steady decline in visual function, while *RPE65* mutations are an improvement type of mutation that are associated with a transient improvement and a subsequent decline in visual function^7^. The course of disease progression in LCA presents a challenge for gene therapy because the retention of sufficient photoreceptors is a prerequisite for a satisfactory therapeutic outcome. In an animal study, mice lacking *RPGRIP1* showed highly disorganized photoreceptor outer segments at 20 days of age and photoreceptor loss by 5 months of age^8^. Accordingly, there is treatment potential for LCA associated with *RPGRIP1* mutations. In a previous study in which gene therapy was performed on a murine model with *RPGRIP1* mutations, ERG and histological examinations showed better preservation of photoreceptor function in the treated eyes^9^. In another study, gene therapy was effective in the early stage of disease in LCA patients with *RPE65* mutations^10^. Although gene therapy can be more effective in patients in the early stage of LCA, to our knowledge, few studies have evaluated the retinal structure in LCA caused by *RPGRIP1* mutations. In the present study, we evaluated the retinal structure in the early stage of LCA caused by *RPGRIP1* mutations.

## Materials and methods

### Ethics statement

This retrospective study was approved by the Institutional Review Board of the Hamamatsu University School of Medicine (permit no. 14–040) and National Center for Child Health and Development (permit no. 686). The study protocol adhered to the principles of the Declaration of Helsinki. Written informed consent was obtained from the parents of all patients before any study procedure or examination was performed.

### Clinical assessments

Four Japanese patients from two families who visited the National Center for Child Health and Development (NCCHD) were included in this study. These patients were diagnosed with LCA as in our previous study^11^. We reviewed the hospital records of all four patients, comprising the clinical findings and general medical and ophthalmic examinations, which included best-corrected visual acuity (BCVA) measurements, slit-lamp biomicroscopy, ophthalmoscopy after pupillary dilation, and Goldmann perimetry (GP) for kinetic visual field assessments. Fundus photography and fluorescein angiography (FA) were performed with the TRC-50LX (Topcon, Tokyo, Japan) and the RetCam imaging system (Natus Medical Inc., Pleasanton, CA, USA). The central retinal laminar architecture was evaluated using spectral-domain optical coherence tomography (SD-OCT; RS-3000, Nidek, Gamagori, Japan) and swept-source OCT (SS-OCT; Topcon). Full-field ERG was performed in accordance with the International Society for Clinical Electrophysiology of Vision protocol^12^. OCT and ERG were performed under general anesthesia.

### Target capture and next-generation sequencing (NGS)

The integrity of the targeted NGS approach used in this study has been previously evaluated^11^. Library preparation for NGS was performed using the HaloPlex Target Enrichment Kit 500 kb (Agilent Technologies, Santa Clara, CA, USA) in accordance with the manufacturer’s instructions. A custom target enrichment library was designed to capture the 74 genes known to be associated with retinitis pigmentosa (RP) or LCA as reported in RetNet at the time of system design^5^. Probes were generated for 1182 regions to cover all exons and flanking intronic sequences (intronic sequence, ±25 bp from the exon boundaries) of the 74 genes. Amplicon libraries were prepared from the genomic DNA of patients in accordance with the manufacturer’s instructions^13^. A DNA sample library was quantified and loaded onto an MiSeq sequencer (Illumina, San Diego, CA, USA) in accordance with the manufacturer’s instructions using 150-bp paired-end chemistry.

### NGS data analysis

All sequence and statistical analyses were performed using relevant programs in the commercially available, stand-alone Genomics Workbench software package (version 8.5.1; CLC bio, Aarhus, Denmark)^13^. We focused on nonsynonymous variants and splice site variants within 5 bp of the exon–intron boundaries (±5 bp) and excluded synonymous and noncoding exonic variants from the analysis. Common genetic variants (allele frequency, >0.005 for recessive variants or >0.001 for dominant variants) in any of the ethnic subgroups found in the following single nucleotide polymorphism (SNP) databases and synonymous variants were treated as possible nonpathogenic sequence alterations: 1000 Genomes database^14^, Exome Aggregation Consortium database^15^, Human Genetic Variation Database (HGVD)^16^, and Tohoku Medical Megabank Organization (ToMMo) database^17^. The HGVD and the ToMMo databases were used as a reference for Japanese controls. The Human Gene Mutation Database^18^ was used to screen for mutations reported in published studies.

### Molecular validation of the candidate variants

Potential pathogenic mutations detected by NGS were validated using standard Sanger sequencing^19^. Sanger sequencing segregation analyses were performed for DNA from family members to investigate the cosegregation of potential pathogenic mutations. The following primer sets were used in the current study: exon 11 in the *RPGRIP1* forward primer 5′-TGGGAAGATTAAATTCACACTTGA-3′ and reverse primer 5′-GTTAGTTTTCTAATCTCATCATCTTCC-3′ and exon 22 in the *RPGRIP1* forward primer 5′-AAAGCAGTTGGTCCATGTTATTCT-3′ and reverse primer 5′- AGGTAATGGATTAGGTAGTCACAAA-3′.

Screening for the known exon 17 deletion mutation in *RPGRIP1*^11,20^ was performed in the patient in case 4, in whom a targeted NGS approach had revealed a single heterozygous *RPGRIP1* mutation. To identify the deletion breakpoints, long-range PCR and direct sequencing analysis were performed using the primers described by Suzuki et al.^20^

## Results

### Clinical findings

The clinical, visual field, and electrophysiological findings are summarized in Table 1.

**Table 1:** Characteristics of the four patients with Leber’s congenital amaurosis (LCA) who were included in the present study

					BCVA	Visual field (V4e)	Electroretinograms		Clinical examination findings					
							Rod	Cone						
Case	Age gender	Nyctalopia	Nystagmus	Oculo digital sign	OD OS	OD OS			Kerato-conus	Cataract	Macular atrophy or staphyloma	Attenuated vessels	Chorioretinal atrophy	Peripheral pigmentation
1 EYE20	7 F	+	+	−	16/200 20/200	5° 5°	Reduced	Not detectable	−	−	−	+	+	+
					(13 years of age)	(12 years of age)	(7 years of age)							
2 EYE64	1 M	+	+	−	12/200 12/200	30° 30°	Subnormal	Not detectable	−	−	−	±	+	±
					(7 years of age)	(7 years of age)	(1 year of age)							
3 EYE65	1 M	+	+	−	4/200 4/200	20° 20°	Subnormal	Not detectable	−	−	−	±	+	±
					(7 years of age)	(7 years of age)	(1 year of age)							
4 EYE55	9 M	+	+	−	30/200 30/200	90° 90°	Subnormal	Not detectable	−	−	−	−	+	−
					(15 years of age)	(15 years of age)	(9 years of age)							

Age at the examination is given in parentheses

**Fig. 1:**
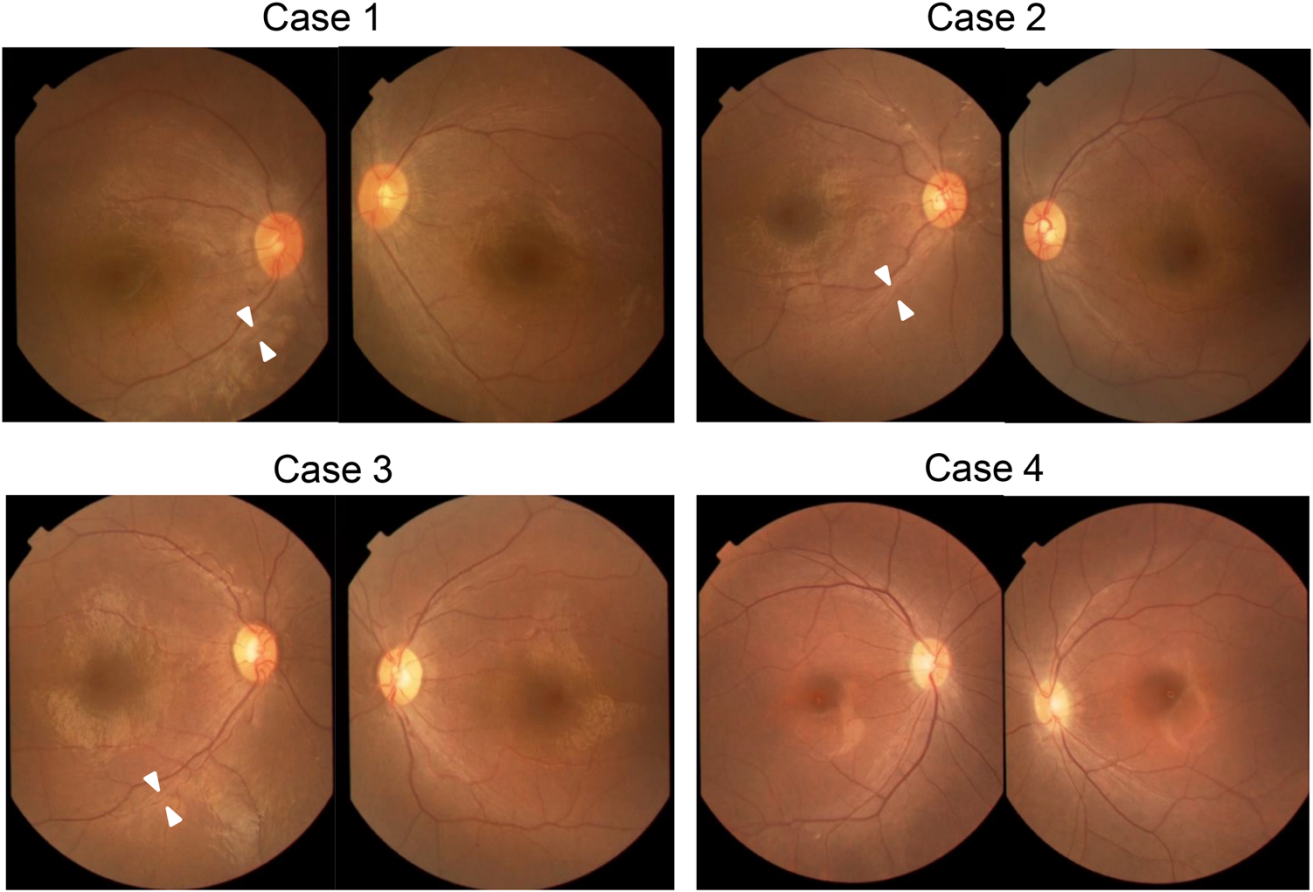
Color fundus photographs of four patients with Leber’s congenital amaurosis (LCA) caused by *RPGRIP1* mutations. Fundus photographs of both eyes show attenuated retinal vessels (arrowheads) in cases 1–3 (EYE20, 64, and 65). In the photographs for case 4 (EYE55), the fundus lacks the typical changes seen in LCA

**Fig. 2:**
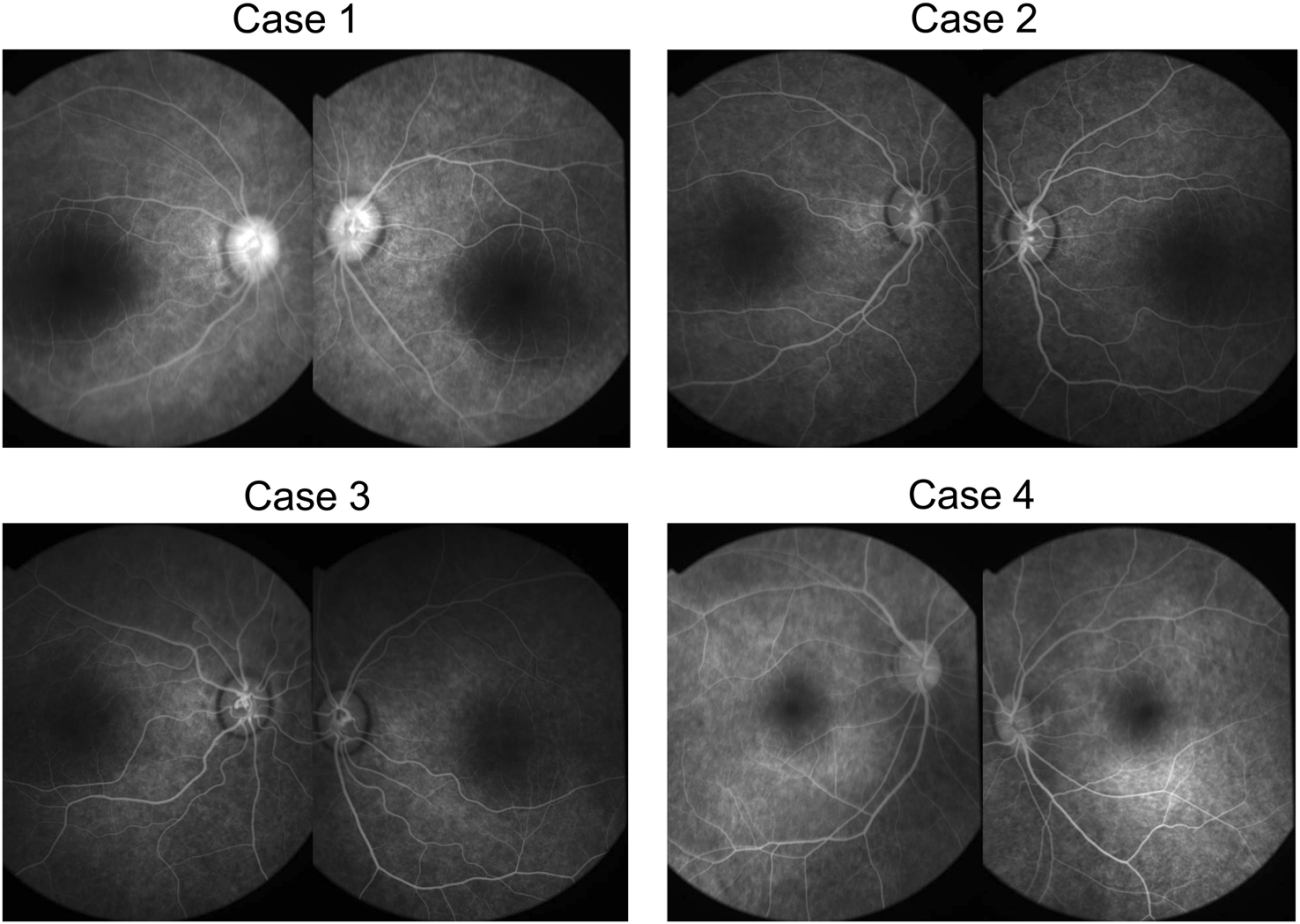
Fluorescein angiographies (FA) of four patients with Leber’s congenital amaurosis (LCA) caused by *RPGRIP1* mutations. FA of both eyes showed granular hyperfluorescence without leakage due to retinal pigment epithelium atrophy in all four patients

**Fig. 3:**
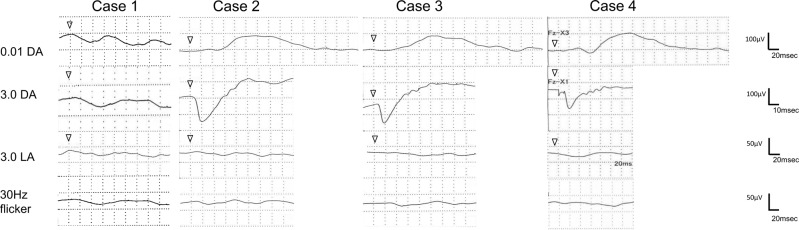
Electroretinograms of four patients with Leber’s congenital amaurosis (LCA) caused by *RPGRIP1* mutations. The rod response is reduced (beginning at 7 years of age) in case 1 (EYE20). In cases 2 and 3 (EYE64 and EYE65, respectively), the rod response is abnormal (beginning at 1 year and 3 months of age). In case 4 (EYE55), the rod response is abnormal (beginning at 7 years of age). The cone responses are not detectable in all 4 cases

**Fig. 4:**
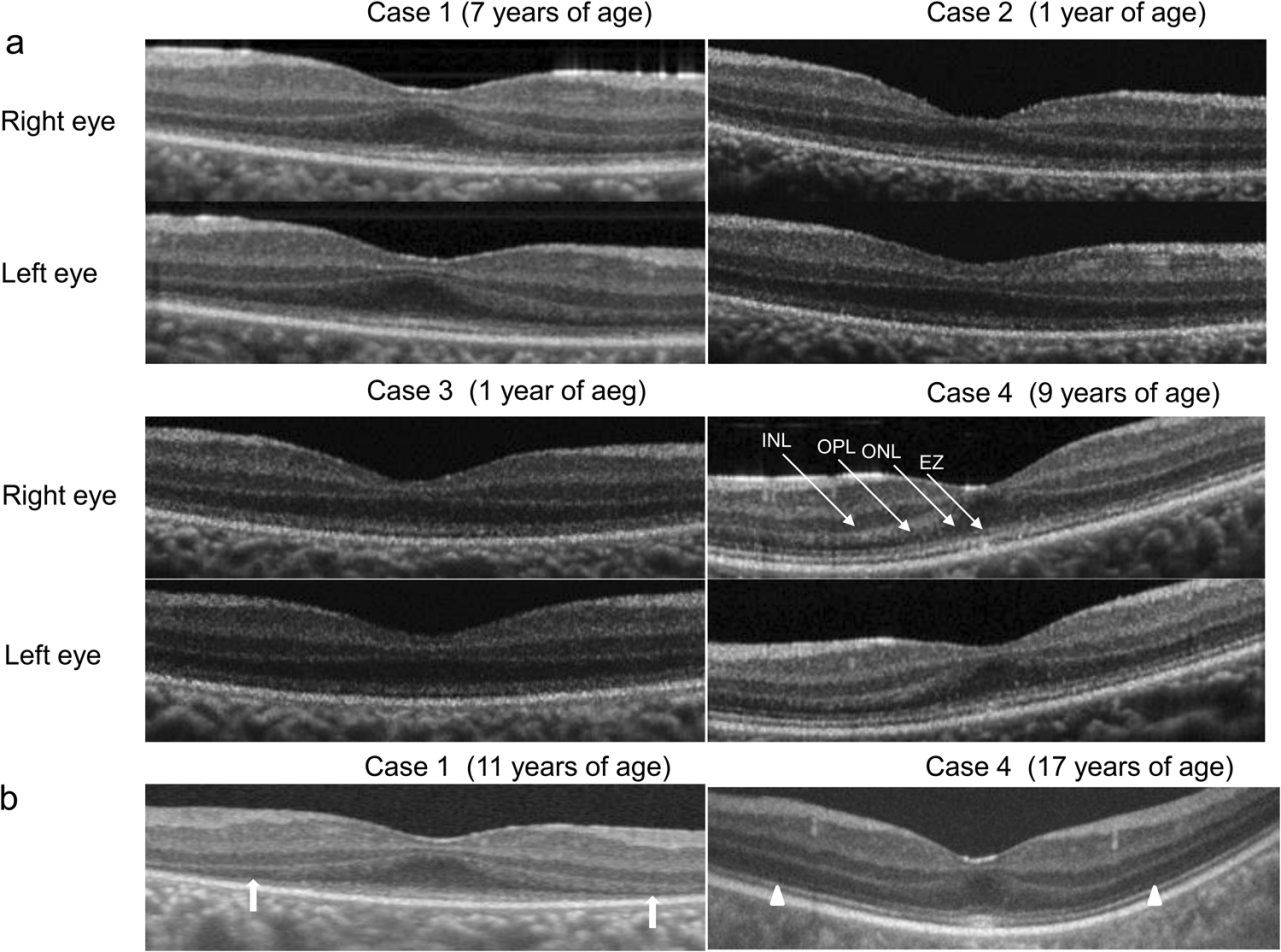
Optical coherence tomography (OCT) findings for four patients with Leber’s congenital amaurosis (LCA) caused by *RPGRIP1* mutations. **a** The lamellar structure in the foveal area is retained in all four patients. In cases 1–3 (EYE20, 64 and 65), the ellipsoid zone is extinguished. In case 4 (EYE55), the ellipsoid zone is retained. Arrows indicate the lamina corresponding to the inner nuclear layer (INL), outer plexiform layer (OPL), outer nuclear layer (ONL) and ellipsoid zone (EZ). **b** OCT for case 1 (EYE20) shows changes in the macular structure with an increase in age. Although a continuous ONL with a distinct adjacent lamellar architecture is detectable in the foveal area on OCT performed at 7 years of age, ONL is not distinct in the perifoveal area (arrows) on OCT performed at 11 years of age. OCT indicates slight ONL thinning between 7 and 11 years of age. In OCT for case 4 (EYE55), the ellipsoid zone is maintained at 9 years of age but is blurred at 17 years of age (arrowheads)

Case 1 (EYE20) was a 13-year-old girl who visited the NCCHD with congenital nystagmus and visual impairment at 7 years of age. Slit-lamp biomicroscopy showed normal findings. On funduscopic examination, both eyes showed retinal degeneration with attenuated vessels, chorioretinal atrophy, and peripheral pigmentation (Figs. 1, 2). At 7 years of age, full-field ERG showed a reduced rod response and an extinguished cone response (Fig. 3), while OCT showed a thinning photoreceptor outer nuclear layer (ONL) with a distinct adjacent lamellar architecture; notably, the ellipsoid zone was extinguished (Fig. 4a). At 11 years of age, ONL was not distinct in the perifoveal region, although lamellar structure was retained in the fovea (Fig. 4b). At 11 years of age, retinal aneurysmatic vessels with focal exudates (located in the inferior temporal mid-periphery) were treated with laser photocoagulation. Goldmann perimetry performed at 12 years of age revealed concentric constriction of the visual field. At 13 years of age, her decimal BCVA was 0.08 (+4.00/+3.00/5°) in the right eye and 0.1 (+4.00/+3.00/5°) in the left eye.

Cases 2 and 3 (EYE64 and 65, respectively) were 7-year-old homozygotic twin boys diagnosed with LCA with congenital nystagmus and visual impairment at 1 year and 3 months of age. Slit-lamp biomicroscopy showed normal findings. On funduscopic examination, both eyes showed retinal degeneration with slightly attenuated vessels, chorioretinal atrophy, and mild peripheral pigmentation (Figs. 1, 2). Full-field ERG performed at 1 year and 3 months of age showed a subnormal rod response and an extinguished cone response (Fig. 3). OCT showed a thinning photoreceptor ONL with a distinct adjacent lamellar architecture at 1 year and 3 months of age; however, the ellipsoid zone was extinguished (Fig. 4a). At 7 years of age, BCVA was 0.06 (+4.00/+1.00/180°) in the right eye and 0.06 (+1.00/+3.00/180°) in the left eye for case 2, and 0.02 (+4.50) in the right eye and 0.02 (+5.50) in the left eye for case 3. Goldmann perimetry performed at 7 years of age showed concentric constriction of the visual field.

Case 4 (EYE55) was a 17-year-old boy diagnosed with LCA with congenital nystagmus and visual impairment at 7 years of age. Slit-lamp biomicroscopy showed normal findings. On funduscopic examination, both eyes appeared normal (Figs. 1, 2). Full-field ERG performed at 9 years of age revealed an extinguished cone response and a subnormal rod response (Fig. 3); OCT performed at 9 years of age showed a thinning photoreceptor ONL with a distinct adjacent lamellar architecture (Fig. 4a). The ellipsoid zone was maintained at 9 years of age but became blurred at 17 years of age (Fig. 4b). His BCVA at 15 years of age was 0.15 (−2.50/−1.50/°) in the right eye and 0.15 (−3.00/−1.50/°) in the left eye. Goldmann perimetry showed low sensitivity within each isopter.

### Findings of targeted NGS and potential pathogenic mutations in the two families

These patients were diagnosed with LCA in our previous study^11^. Findings of targeted NGS are shown in the Supplementary Information and Supplementary Table 1. The obtained sequence data were analyzed using a previously described bioinformatics pipeline^13^. Cases 1–3 exhibited the homozygous deletion mutation c.3565_3571delCGAAGGC, whereas case 4 exhibited a heterozygous splicing mutation c.1467+1G>T and the heterozygous deletion mutation c.2710 + 374_2895 + 78del (Supplementary Figure, Supplementary Table 2, Supplementary Information)^11^.

## Discussion

In the present study, we evaluated the retinal structure in four young patients with LCA caused by *RPGRIP1* mutations. All patients in this study exhibited congenital nystagmus, poor visual acuity, and photophobia, which are characteristic of LCA. Cases 1–3 exhibited attenuated vessels, chorioretinal atrophy, and peripheral pigmentation; conversely, case 4 exhibited a normal fundus. Thus, these patients exhibited various, but not distinct, clinical findings compared with previously reported patients with *RPGRIP1*-associated LCA^1,3,7,11,20–22^. For example, although dysfunctions of both rod and cone photoreceptors are characteristic features of LCA, ERG on *RPGRIP1*-associated LCA is known to have a broader spectrum; Suzuki et al. described a patient who showed cone-dominant affected ERG^20^.

A few reports using OCT have mentioned that the central retinal laminar architecture, including the photoreceptor cell layer, is preserved in teenagers with *RPGRIP1*-associated LCA^22^. Wang et al. reported an abnormal macular structure identified using OCT at 5 years of age in a patient with *RPGRIP1*-associated LCA. Their OCT images demonstrated a relatively preserved foveal lamellar structure, decreased ONL thickness, and hardly visible ellipsoid zone band^22^. We evaluated the retinal structure on OCT in patients with *RPGRIP1*-associated LCA before adolescence; in particular, cases 2 and 3 were examined at 1 year and 3 months of age. To our knowledge, there have been no reports regarding OCT at such an early stage of *RPGRIP1*-associated LCA. The findings for cases 2 and 3 suggest that the ONL is better retained in early childhood than at 5 years of age^22^. In case 1, the ONL layer in the parafoveal area appeared thinner at 11 years of age compared with 7 years of age. In addition, in case 4, the ellipsoid zone was maintained at 9 years of age but became blurred at 17 years of age. These findings suggested that gene therapy in early childhood may provide better benefit to LCA patients with *RPGRIP1* mutations.

*RPGRIP1* is located on chromosome 14q11. It consists of 24 exons and encodes a protein with 1287 amino acids. The RPGRIP1 protein contains several structurally conserved motifs, a coiled coil (C2) domain, an RCC homology domain known as the RPGR interacting domain (RID), and a domain of unknown function^23^. RPGRIP1 interacts with RPGR in the cilium connecting the outer and inner photoreceptor segments, plays a role in ciliary trafficking in support of outer segment morphogenesis, and promotes outer segment development^24^. In addition, multiple proteins have synergistic actions; RPGRIP1 is necessary for RPGR binding to CEP290^25^, NPHP4 interacts with RPGRIP1 via a C2 domain^26^, and SPATA7 also interacts with RPGRIP1 via a C2 domain^27^. The loss of *RPGRIP1* leads to the loss of RPGR in the connecting cilia. In a previous study, the loss of *RPGRIP* in mice resulted in a grossly oversized outer segment disc morphology^8^.

The homozygous deletion mutation c.3565_3571delCGAAGGC was detected in cases 1–3 in the present study. These patients exhibited phenotypic features similar to those already reported for *RPGRIP1*-associated LCA; a Korean patient with LCA who had the compound heterozygous deletion mutation c.3565_3571delCGAAGGC and missense mutation c.1892A>T showed photophobia and peripheral hyperpigmentation^28^. Case 4 exhibited a compound heterozygous splicing mutation c.1467+1G>T and an exon 17 deletion mutation. Suzuki et al. reported two Japanese brothers (7 and 11 years of age) with homozygous exon 17 deletion mutations in *RPGRIP1*; both were diagnosed with LCA and exhibited congenital nystagmus and visual impairment. They had a normal fundus and demonstrated recordable single-flash ERGs and undetectable 30-Hz flicker ERGs^20^. The c.1467+1G>T mutation was located in the intron 11 donor site. To predict the effect of the nucleotide substitution in the splice sites on splicing, we performed in silico analysis using splice site prediction^29^. The splice donor site score of the normal allele was 1.00, suggesting a high enough ability for splicing (score range of 0‒1 with a larger score indicating a greater ability), but the mutant allele was not recognized as a splicing donor site. Therefore, our results suggest that the c.1467+1G>T mutation causes intron 11 to remain in the transcript and undergo translation. This would produce a stop codon with 32 amino acids downstream, possibly resulting in nonsense-mediated mRNA decay (NMD)^30^. The splicing mutation c.1467+1G>T, resulting in a nonprotein product, and the lack of exon 17 would be a truncating mutation. Huang et al. suggested a correlation between the disease severity and the nature of the mutations in three patients with retinal dystrophy associated with *RPGRIP1* mutations. The patient with the homozygous mutation ex1–22del was considered to have a severe form of the disease. The patient with the homozygous splicing mutation c.1468–2A>G, which is an in-frame mutation, was considered to have a less severe form of the disease, and the patient with the compound heterozygous nonsense mutation c.154C>T and missense mutation c.2020C>T was considered to have a less severe form of the disease^31^. According to that study, truncating mutations appeared to result in a more severe disease phenotype than did missense or in-frame splicing mutations. *RPGRIP1* mutations result in retinal dystrophies with a broad range of phenotypes, ranging from LCA as a severe form to cone–rod dystrophy as a less severe form. In the present study, truncating mutations were detected in all four patients who exhibited characteristic LCA phenotypes.

This study is limited by the small number of patients. Further genetic analysis of a larger sample size and accumulation of clinical findings are necessary to understand the genotype–phenotype correlation in patients with *RPGRIP1*-associated LCA.

In conclusion, we described the features of young patients with LCA caused by *RPGRIP1* mutations and evaluated retinal structure at a very early stage in patients with *RPGRIP1*-associated LCA. Although all four patients exhibited various clinical features, our findings suggest that the ONL on OCT is relatively well preserved in the early stage of *RPGRIP1*-associated LCA. Further analyses are needed to further clarify our findings.

## Supplementary information

Supplementary Information

Supplementary Table 1

Supplementary Table 2

Supplementary Figure
