# Supplementary material for: Retinal structure in Leber’s congenital amaurosis caused by RPGRIP1 mutations
Source: Hum Genome Var. 2019 Jun 27;6:32. doi: 10.1038/s41439-019-0064-8 (PMC6804879; doi:10.1038/s41439-019-0064-8)
Supplement: Supplementary file 1 — Supplementary Information [file 41439_2019_64_MOESM1_ESM.docx]

**Retinal structure in the Leber’s congenital amaurosis caused by *RPGRIP1* mutations**

Daisuke Miyamichi^1^, Sachiko Nishina^2^, Katsuhiro Hosono^1^, Tadashi Yokoi^2^, Kentaro Kurata^1^, Miho Sato^1^, Yoshihiro Hotta^1^, Noriyuki Azuma^2^

**^1^** Department of Ophthalmology, Hamamatsu University School of Medicine, Shizuoka, Japan

^2^ Department of Ophthalmology and Laboratory for Visual Science, National Center for Child Health and Development, Tokyo, Japan

**Supplementary Information**

**Findings of targeted NGS**

Each target capture panel comprised 445,968 bp derived from the 1182 target regions of the 74 genes. Target region-enriched DNA was sequenced using NGS. The mean number of generated reads was 2,137,883, and approximately 89% of these were mapped to targeted regions, which exhibited an average 272.8 ± 62.8-fold coverage in the four samples. In addition, an average of 93.2% and 87.7% bases in the target region exhibited 20-fold and 40-fold coverage, respectively. These results indicate that sufficient coverage for the identification of variants was achieved. Data for each of the four patients are shown in Supplementary Table 1.

**Potential pathogenic mutations in the two families**

The obtained sequence data were analyzed using a previously described bioinformatics pipeline.^13^ Following the exclusion of common variants (allele frequency, >0.005 for recessive variants and >0.001 for dominant variants) and synonymous variants, one rare homozygous (c.3565_3571delCGAAGGC) and one rare heterozygous (c.1467+1G>T) *RPGRIP1* variant remained in cases 1-3 and case 4, respectively. *RPGRIP1* was indicated as a causative gene for autosomal recessive LCA or autosomal recessive cone-rod dystrophy. Large deletion or insertion mutations were not detectable by the current approach; therefore, screening for the known exon 17 deletion mutation in *RPGRIP1*^11,20^ was performed using long-range PCR in case 4. To identify the deletion breakpoints, direct sequencing analysis was performed using the primers described by Suzuki et al.^20^ As a result, we detected a heterozygous 1339-bp deletion mutation involving exon 17 (c.2710+374_2895+78del). Thus, cases 1-3 exhibited the homozygous deletion mutation c.3565_3571delCGAAGGC, whereas case 4 exhibited a heterozygous splicing mutation c.1467+1G>T and the heterozygous deletion mutation c.2710+374_2895+78del. These c.3565_3571delCGAAGGC, c.2710+374_2895+78del and c.1467+1G>T mutations have been reported as causative mutations for autosomal recessive LCA.^11,20^ Multi-gene LCA cases caused by the mutations c.3565_3571delCGAAGGC and c.2710+374_2895+78del have been reported,^11,20^ whereas the c.1467+1G>T mutation have been reported in one case (Supplementary Table 2).^11^ The results of Sanger sequencing confirmed the mutations. In addition, the mutations were found to co-segregate with the disease phenotype (Supplementary figure).

**Supplementary Figure. Pedigree details of the *RPGRIP1* gene mutations.**

A. Pedigree of Cases 1-3. Case 1 was a 13-year-old girl (II-1), cases 2 and 3 were 7-year-old monozygotic twin brothers (II-2 and II-3, respectively). Cases 1-3 exhibited the homozygous deletion mutation c.3565_3571delCGAAGGC.

B. Pedigree of Case 4 (II-1). Case 4 was a 15-year-old boy. Case 4 exhibited a heterozygous splicing mutation c.1467+1G>T and the heterozygous deletion mutation c.2710+374_2895+78del. Their father (I-1) and mother (I -2) were heterozygous carriers of c.1467+1G>T and c.2710+374_2895+78del, respectively.

Squares indicate males and circles indicate females. Filled symbols indicate individuals affected by Leber congenital amaurosis. The probands are indicated with arrows.
