# Supplementary material for: Retinal structure in Leber’s congenital amaurosis caused by RPGRIP1 mutations
Source: Hum Genome Var. 2019 Jun 27;6:32. doi: 10.1038/s41439-019-0064-8 (PMC6804879; doi:10.1038/s41439-019-0064-8)
Supplement: Supplementary file 2 — Supplementary Table 1 [file 41439_2019_64_MOESM2_ESM.pdf]

Supplementary table 1

Family ID	Number of target region	Total length of target regions (bp)	Total mapped reads	Mapped reads in targeted region	Specificity (%)	Average coverage	Fraction of target coverd with at least 1x (%)	Fraction of target coverd with at least 20x (%)	Fraction of target coverd with at least 40x (%)
EYE20 (II-1)	1,182	445,968	1,989,867	1,744,502	87.67	249.1	98.2	93.64	88.71
EYE64 (II-2)	1,182	445,968	2,590,090	2,334,130	90.12	337.9	98.21	94.39	90.43
EYE65 (II-3)	1,182	445,968	1,876,263	1,682,569	89.68	238.1	97.9	89.98	81.56
EYE55 (II-1)	1,182	445,968	2,095,311	1,834,254	87.54	266.1	98.32	94.72	90.05
