# Supplementary material for: Retinal structure in Leber’s congenital amaurosis caused by RPGRIP1 mutations
Source: Hum Genome Var. 2019 Jun 27;6:32. doi: 10.1038/s41439-019-0064-8 (PMC6804879; doi:10.1038/s41439-019-0064-8)
Supplement: Supplementary file 3 — Supplementary Table 2 [file 41439_2019_64_MOESM3_ESM.pdf]

Supplementary table 2

Gene	Accession number	Eye ID	Mutation type	Nucleotide change	Predicted effect	Location in gene	Genotype	SNP databases				Reference
								ExAC	1000 Genomes	HGVD	ToMmo	
RPGRIP1	NM_020366.3	EYE20 (II-1)	Deletion	c.3565_3571delCGAAGGC	p.(R1189Gfs*7)	Exon 22	Homozygous	1.66×10^{-4}	0	0	0	11, 28
		EYE64 (II-2)	Deletion	c.3565_3571delCGAAGGC	p.(R1189Gfs*7)	Exon 22	Homozygous	1.66×10^{-4}	0	0	0	11, 28
		EYE65 (II-3)	Deletion	c.3565_3571delCGAAGGC	p.(R1189Gfs*7)	Exon 22	Homozygous	1.66×10^{-4}	0	0	0	11, 28
		EYE55 (II-1)	Splice	c.1467+1G>T		Intron 11	Compound Heterozygous	0	0	0	0	11
			Deletion	c.2710+374_2895+78del		Exon 17	Compound Heterozygous	0	0	0	0	11, 20

The nucleotide numbering reflects cDNA numbering with +1 corresponding to A of the ATG translation initiation codon in the reference sequence NM_020366.3.
