# Supplementary figures and images for: Retinal structure in Leber’s congenital amaurosis caused by RPGRIP1 mutations
Source: Hum Genome Var. 2019 Jun 27;6:32. doi: 10.1038/s41439-019-0064-8 (PMC6804879; doi:10.1038/s41439-019-0064-8)

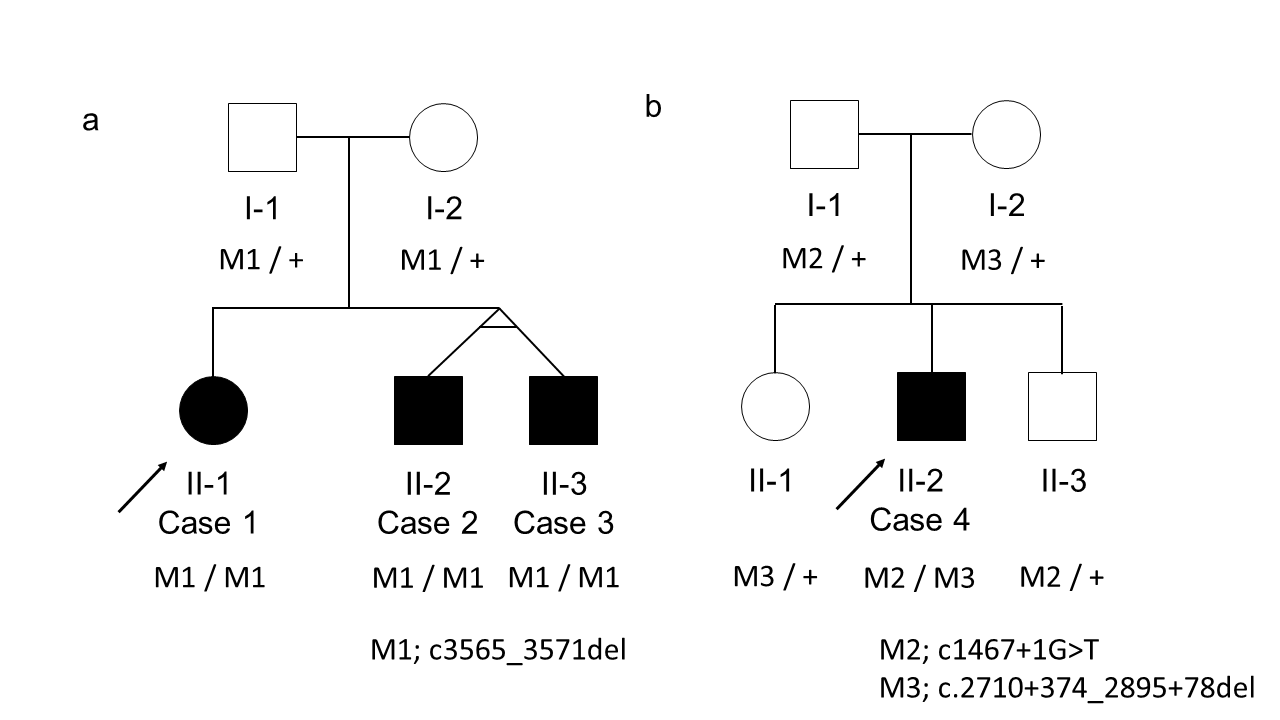

Supplement: Supplementary file 4 — Supplementary Figure [file 41439_2019_64_MOESM4_ESM.tif]
